# Clinical features of pediatric post-acute COVID-19: a descriptive retrospective follow-up study

**DOI:** 10.1186/s13052-021-01127-z

**Published:** 2021-08-26

**Authors:** Liene Smane, Ieva Roge, Zanda Pucuka, Jana Pavare

**Affiliations:** 1 Children’s Clinical University Hospital, Riga, Latvia; 2 Department of Paediatrics, Riga Stradins University, Riga, Latvia

**Keywords:** SARS-CoV-2, Post-acute COVID-19, Pediatrics

## Abstract

To date, information on COVID-19 long-term post-recovery sequelae in children and adolescents remains scarce. A retrospective descriptive cohort study was performed by collecting data on 92 patients (age ≤ 18 years). All were evaluated during a face-to-face visit following a specially designed post-COVID-19 symptom assessment protocol at the following stage: 1–3 months after COVID-19 onset. Among the 92 children, 45 (49%) were completely free of any COVID-19-related symptoms, while 47 (51%) reported persistence of at least one symptom, in particular tiredness, loss of taste and/or smell and headaches. The most common post-acute COVID-19 clinical features were noted in children aged between 10 and 18 years. A detailed multidisciplinary follow-up of patients with COVID-19 seems relevant, whatever the severity of the symptoms.

To the Editor,

Severe acute respiratory syndrome coronavirus 2 (SARS-CoV-2) infection causes a spectrum of characteristics ranging from asymptomatic seroconversion to severe coronavirus cases, sometimes with prolonged symptoms [1]. Current data shows that prolonged symptom duration is common not only in adults with severe and non-severe coronavirus disease 2019 (COVID-19), but also among children with COVID-19 [2–7]. Post-acute COVID-19 clinical features vary widely. The most commonly reported persistent symptoms after recovery from COVID-19 among the symptomatic outpatients were insomnia, fatigue, coughing, dyspnea, loss of taste and/or smell and headaches [3, 5–7]. As noted by Hoang et al., SARS-CoV-2 infection in children has a milder clinical course commonly characterised by fever, cough, rhinorrhea and myalgia/fatigue [8]. This contrasts with adults, characterising a return to baseline health among children with milder COVID-19, although the clinical course is not sufficiently described.

Children’s Clinical University Hospital in Riga established a post-acute outpatient follow-up service for individuals who had recovered from COVID-19. Before the start of December 2020, 3509 children were diagnosed with SARS-CoV-2 infection, representing approximately 8.6% of all COVID-19 patients in Latvia. We conducted a retrospective cohort study, including 92 COVID-19 disease outpatients (age ≤ 18 years) and their caregivers (parents or legal family representatives) from March 12, 2020 (the date COVID-19 was declared a state of emergency in Latvia), to December 1, 2020. A real-time polymerase chain reaction (RT-PCR) for SARS-CoV-2 was used, and the study included children with a negative test result. Patients were tested systematically for viral co-infections. Caregivers had to provide written consent for this. To identify the long-term consequences of SARS-CoV-2 infection, all patients enrolled in the study were evaluated by two pediatricians in a face-to-face visit according to a specially designed post-COVID-19 symptom assessment protocol (supplementary material) developed based on previously published literature about Long COVID in adults [2, 3, 9, 10]. Post-COVID-19 evaluation protocol consists of the following domains: physical and mental health, social and psycho-emotional wellbeing. During the follow-up, we collected demographic and epidemiological characteristics, vaccination status, baseline chronic medical conditions and clinical features during the acute phase of SARS-CoV-2, as well as the clinical symptoms that were persistent at the time of the first visit. We collected this information in a structured questionnaire (which medical staff filled in). For this data analysis, we excluded patients who had not consented to the follow-up visits or who could not be reached, as well as those who showed no symptoms at testing, or those in whom co-infections were diagnosed. Descriptive statistics were used to present the data. The ethics committee of Riga Stradins University and the Institutional Review Board of Children’s Clinical University Hospital (No. 6–1/07/35) reviewed and approved the study protocol questionnaire and informed consent forms.

We invited 189 patients who had been tested in an ambulatory setting to participate in the study by telephone, with 92 face-to-face follow-up first visits completed. Among the 189 patients, we excluded 62 from the study who could not be reached, 27 who were not willing to participate in the study and 8 who did not answer the questions about symptoms. No co-infections were observed in patients of the study group. The median age of the study group was 12 years (interquartile range [IQR] = 8–15 years), 56 (61%) were boys. Overall, 18 children reported chronic medical conditions. The median interval from the test to the first follow-up visit was 55 days (IQR = 30–104 days). Among all 92 outpatients during the acute phase of SARS-CoV-2 infection, tiredness was the most commonly reported symptom in 42 (46%) and fever in 39 (42%), with headaches in 34 (37%), while a cough was present in 30 (33%) patients. Fever was defined as an axillary temperature of 37.5 °C or higher. The characteristics of the study population are summarised in Table 1. At the time of the follow-up visit, 45 (49%) were completely free of any COVID-19-related symptoms, while 19% had 1 symptom, 10% had 2, and 22% had 3 or more. We have not looked at the correlation between the persistence of symptoms and distance from the acute phase of the infection. The Figure shows that patients still commonly reported tiredness (38%), loss of taste and/or smell (16%), and headaches (15%) (Fig. 1). Sensory disturbances (photophobia, sound sensitivity) occurred in 10 (11%) patients, while cognitive disturbances (memory, attention, and information processing problems) were present in 9 (10%). Other less common symptoms of the cohort are displayed in Table 2.

**Fig. 1:**
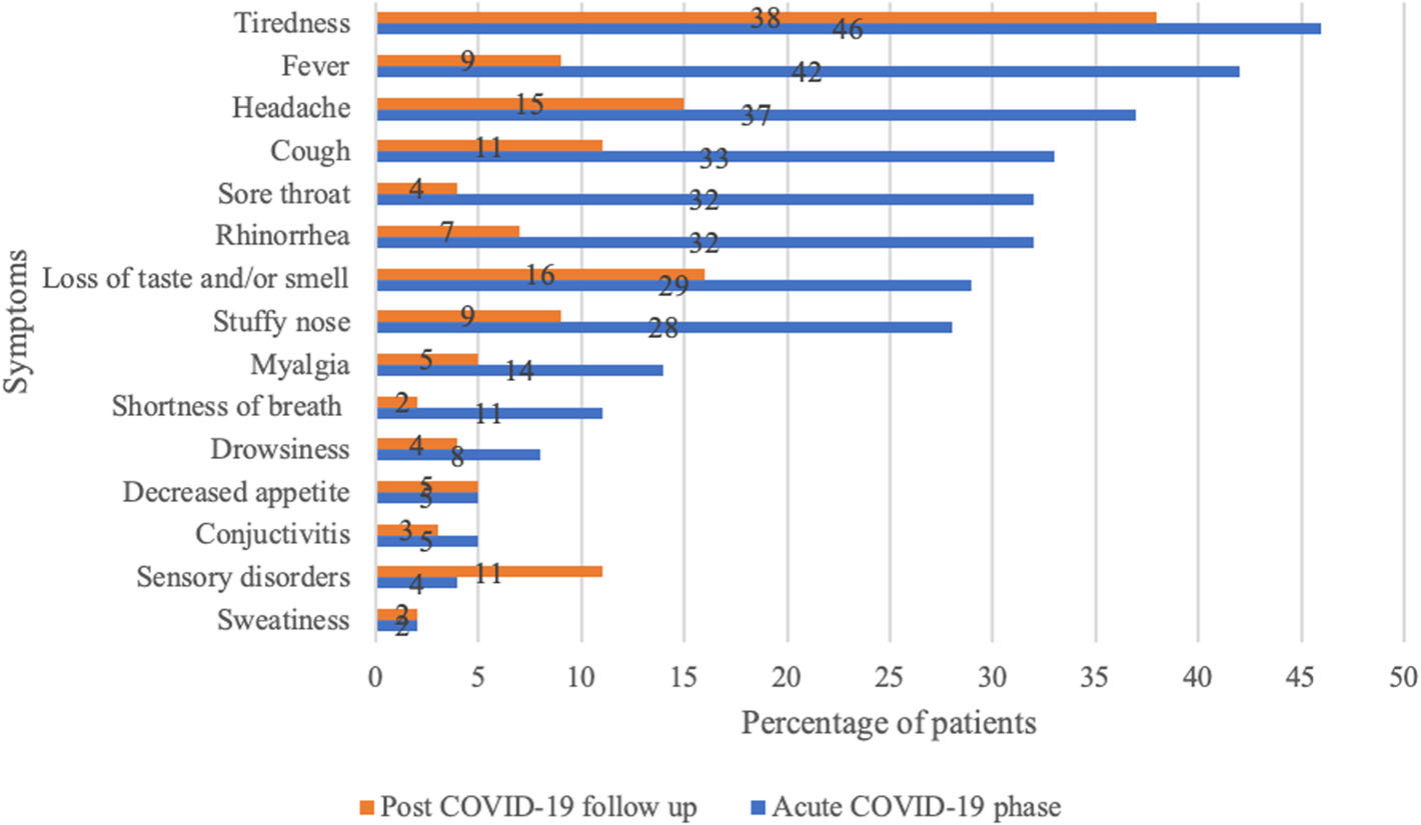
COVID-19-related symptoms in children during the acute phase of the disease and at the time of the follow-up visit

**Table 1:** Characteristics of Symptomatic Children’s COVID-19 Cases (*N* = 92)

Characteristics	Value
**Median (interquartile range) age, years**	12 (8–15)
**Age group, No. (%):**	
1 month to < 1 year	None
1–4 years	10 (11%)
5–9 years	21 (23%)
10–14 years	35 (38%)
15–18 years	26 (28%)
**Sex at birth, No. (%):**	
Male	56 (61%)
Female	36 (39%)
**Vaccination, No. (%)**	
Seasonal influenza	12 (13%)
Complete vaccination	85 (92%)
Incomplete vaccination	12 (13%)
**Total baseline chronic medical conditions, No. (%)**	18 (20%)
Bronchial asthma	7 (8%)
Attention-deficit/hyperactivity disorder	3 (3%)
Atopic dermatitis	2 (2%)
Arthritis	1 (1%)
Patent ductus arteriosus	1 (1%)
Vegetative dystonia	1 (1%)
Congenital hypothyroidism	1 (1%)
Epilepsy	1 (1%)
Overactive bladder	1 (1%)
**Exposure to the source of transmission, No. (%)**	
Contact with COVID19 positive patients within the last 14 days	66 (72%)
Contact with group COVID-19 cases	35 (38%)
Contact with a person with fever / respiratory symptoms or epidemiological communities in the last 14 days	22 (24%)
Contact with a person who does not know the COVID-19 test results within 14 days	18 (20%)
Traveling in the last 14 days	17 (18%)
Patients from a care center	2 (2%)
**Acute COVID-19 symptoms, No. (%)**	
Distribution of temperature, No. (%)	
37.2–37.5 °C	36 (39%)
≥ 37.5–38 °C	15 (16%)
38.1–39.0 °C	13 (14%)
> 39.0 °C	11 (12%)
Tiredness	42 (46%)
Headache	34 (37%)
Cough	30 (33%)
Rhinorrhea	29 (32%)
Sore throat	29 (32%)
Loss of taste and/or smell	27 (29%)
Stuffy nose	26 (28%)
Sneezing	22 (24%)
Diarrhea	17 (18%)
Myalgia	13 (14%)
Shortness of breath	10 (11%)
Drowsiness	7 (8%)
Decreased appetite	5 (5%)
Conjunctivitis	5 (5%)
Wheezing	5 (5%)
Vomiting	4 (4%)
Sensory disturbances	4 (4%)
Sweatiness	2 (2%)
Epistaxis	2 (2%)
Periorbital edema	1 (1%)
Seizures	1 (1%)
**Post–acute COVID-19 follow-up characteristics**	
Days since symptoms onset, median (interquartile range)	55 (30–104)
Persistent symptoms, No. (%)	
None	45 (49%)
1	18 (19%)
2	9 (10%)
≥ 3	20 (22%)

**Table 2:** Post-COVID-19 follow-up clinical features

Symptoms	Value	%
Tiredness after sleep	18	20
Persistent fatique	17	18
Loss of taste and/or smell	15	16
Headache	14	15
Sensory disturbances	10	11
Cognitive disturbances	9	10
Prolonged fever	8	9
Stuffy nose	8	9
Joint pain	7	8
Dizziness	7	8
Chest pain	6	7
Cough	6	7
Dry mouth	6	7
Rhinorrhea	6	7
Decreased appetite	5	5
Myalgia	5	5
Orthostatic intolerance	4	4
Sore throat	4	4
Drowsiness	4	4
Red eye syndrome	3	3
Dry eyes	2	2
Shortness of breath	2	2
Sweatiness	2	2
Swollen lymph glands	2	2
Weight loss	1	1
Exercise intolerance	1	1
Microhematuria	1	1

To understand the frequency and nature of long-term complications and persistent symptoms, we are continuing our study and following the study design, which consists of outpatient follow-up visits (at 1, 3, 6, 12, and 24 months after COVID-19 onset) at which several domains are evaluated according to a special questionnaire: physical, mental, social and wellbeing. Researchers have shown that even among young adults aged 18–34 years with no chronic medical conditions, nearly one in five reported prolonged COVID-19-related symptoms [3]. The most commonly reported were fatigue, loss of taste and/or smell and headaches [3, 5]. Our study represents the clinical features of paediatric post-acute COVID-19, with tiredness, loss of taste and/or smell, and headaches among those most commonly reported in outpatients. Previous studies in children found similar presenting symptoms, but they were more common in hospitalized patients [6, 7]. According to our data, the most common post-acute COVID-19 clinical features were noted in children from 10 to 18 years. In conclusion, future studies assessing organ damage will be needed to better understand and characterise Long COVID in children [11]. This study has several limitations. Firstly, this was a retrospective study. Secondly, this study includes a small sample size of patients. Thirdly, the absence of a control group made a comparison with one impossible.

In conclusion, up to 1–3 months after symptom onset, 51% of children with COVID-19 had complaints, mainly tiredness, loss of taste/smell and headaches. A prolonged multidisciplinary follow-up of patients with COVID-19 seems relevant, whatever the severity of the symptoms.

## Data Availability

All data generated or analysed during this study are included in this published article.

## Abbreviations

SARS-CoV-2: Severe acute respiratory syndrome coronavirus 2
COVID-19: Coronavirus disease 2019
